# Anxiety Regulation: From Affective Neuroscience to Clinical Practice

**DOI:** 10.3390/brainsci10110846

**Published:** 2020-11-12

**Authors:** Alessandro Grecucci, Hüseyin Sığırcı, Gaia Lapomarda, Letizia Amodeo, Irene Messina, Jon Frederickson

**Affiliations:** 1 Department of Psychology and Cognitive Sciences, Clinical and Affective Neuroscience Lab, University of Trento, 38068 Rovereto, Italy; sigirci@protonmail.com (H.S.); gaia.lapomarda@unitn.it (G.L.); Letizia.Amodeo@studenti.unitn.it (L.A.); 2 Department of Psychiatry and Psychotherapy III, University of Ulm, 89069 Ulm, Germany; irene-messina@hotmail.com; 3 Washington School of Psychiatry, 5028 Wisconsin Ave NW, Washington, DC 20016, USA; jf1844@gmail.com

**Keywords:** anxiety, psychotherapy, emotion regulation, emotion, psychodynamic

## Abstract

According to psychoanalysis, anxiety signals a threat whenever a forbidden feeling emerges. Anxiety triggers defenses and maladaptive behaviors, thus leading to clinical problems. For these reasons, anxiety regulation is a core aspect of psychodynamic-oriented treatments to help clients. In the present theoretical paper, we review and discuss anxiety generation and dysregulation, first from a neural point of view, presenting findings from neuroimaging and psychophysiological studies. The aim is to trace parallels with psychodynamic theories of anxiety. Then, we discuss the psychological mechanisms and neural bases of emotion regulation in the laboratory, and possible neurobiological mechanisms of anxiety regulation in psychotherapy. We describe two different approaches to emotion/anxiety regulation, one based on the standard cognitive model of emotion regulation, the other based on psychodynamic principles and affective neuroscience. We then illustrate in detail a dynamic experiential approach to regulation. This model claims that emotions arise before cognition and are not inherently dysregulated. Dysregulation emerges from co-occurrences of emotions and associated anxiety. Technical consequences of this model are discussed and include strategies to regulate anxiety.

## 1. The Psychodynamic of Anxiety Generation and Its Neural Bases

Psychodynamic theory makes a distinction between fear and anxiety and between external and internal dangers. When a real external danger is encountered, fear arises [1] to help us to escape from threats. When an internal or imagined danger is experienced, anxiety rises. When an individual experiences an emotion previously perceived as threatening in past relationships and thus associated with an interpersonal danger, anxiety arises [2,3,4,5]. Hence, anxiety is a signal of an arising emotion, which potentially threatens the relationship [1]. During development, children learn which feelings they may feel and express and which ones make their caregivers anxious [2,6]. Feelings that make caregivers anxious may endanger a relationship the child needs to survive [7,8,9]. Children learn to hide emotions that could damage the relationship [6,10]. Hence, anxiety signals that a forbidden feeling is rising [11]. Anxiety occurs outside of patients’ awareness because it is generated non-consciously [4,12]. This is confirmed by neurobiological models of anxiety-generating circuits. Indeed, the autonomic and somatic nervous systems generate the bodily symptoms of anxiety. These systems are activated primarily by the amygdaloidal complex (including the amygdala and the bed nucleus of stria terminalis), hypothalamus, (parahippocampal and cingulate gyri, and the inferior thalamic nuclei), septal nuclei, and portions of the basal ganglia [13]. The prefrontal cortex, the bed nucleus of the stria terminalis, the locus coeruleus, and periaqueductal gray matter are also part of the anxiety circuit. Notably, the prefrontal cortex may be involved at later stages, although not necessarily, and it has been hypothesized to be responsible for the awareness of stimulus-generating anxiety. As predicted by psychodynamic theory, emotions are generated as implicit (unconscious) automatic responses. We now know that the amygdala is responsible for generating anxiety outside of awareness by automatically evaluating the valence of the stimulus [14,15,16]. The amygdala includes the anterocentral, medial, and basolateral portions. The first subgroup shares connections with the hypothalamus and the brain stem, including the solitary tract nucleus and the parabrachial nucleus [14,15,16]. The basolateral group is linked with both the medial and prefrontal orbital cortex within the frontal lobe, as well as the associative cortex within the anterior temporal lobe. These cortico-amygdalar connections play a very important role for two mechanisms hypothesized by psychodynamic theory. First, top-down frontal control of the amygdala can serve as a neural mechanism to regulate anxiety, thanks to the intervention of defense mechanisms (e.g., suppression in dynamic terms). Such top-down modulation has been largely confirmed in emotion regulation neuroimaging experiments (see later sections of this paper). Moreover, a recent study showed that stimulating neural projections to the basolateral amygdala (BLA) may lead to anxiety reduction [17]. These prefrontal-amygdalar connections may be responsible for the understanding, categorization, and symbolization of emotion or stimuli generating anxiety [4].

Psychodynamic theory has always hypothesized that emotions that can harm relationships can trigger anxiety [2]. In other words, psychoanalysis hypothesized a direct link between “dangerous” emotions and anxiety reactions (see the psychodynamics of panic attacks according to dynamic modern treatments [18]). We now know that the bed nucleus of the stria terminalis (BNST) plays a significant role in linking emotional stimuli to anxiety. This region is connected to the amygdala, hypothalamus, but also to hippocampus, prefrontal cortex, locus coeruleus, hypothalamus, raphe nuclei, periaqueductal gray matter, and lateral septum. The BNST can be functionally categorized into the oval (ovBNST), ventral (vBNST), and anterodorsal (adBNST) portions. Exciting the adBNST or suppressing the ovBNST diminishes anxiety. On the contrary, inhibiting the adBNST and exciting the ovBNST may contribute to states of anxiety [19]. Notably, these activations may happen in the absence of awareness, creating a cascade of physiological reactions without awareness, as always hypothesized by psychoanalysis. In the absence of threatening stimuli, it is also possible that the adBNST inhibits the ovBNST, or that the adBNST is not inhibited because the ovBNST is not activated. In fact, inhibition of BLA neurons that are linked to the adBNST provoke anxious behavior. Contrariwise, their activation decreases anxiety. The network of the adBNST, together with the parabrachial nucleus, mediates autonomic responses of anxiety [19]. vBNST carries glutamatergic and excitatory synapses as well as GABAergic and inhibitory synapses with non-dopaminergic cells in the ventral tegmental area (VTA) [19]. Notably, glutamatergic excitatory inputs provoke anxiety and avoidance [20]. This may be the substrate of the defense mechanisms of emotional avoidance as hypothesized by dynamic theorists: if emotions are perceived as too dangerous, the vBNST may help us to avoid situations or stimuli-generating anxiety. Last, but not least, as predicted by early psychoanalytic theorists [1], anxiety can also be a stable personality trait in addition to being a phasic reaction to internal stimuli perceived as dangerous. In a recent study, Saviola and colleagues [5] were able to separate trait from state anxiety. Trait anxiety, defined as a stable personality trait, was associated with both structural covariance of the default mode network (DMN), with an increase in dorsal nodes and a decrease in its ventral part, whereas state anxiety (related to phasic anxious reactions) was widely related to resting state functional connectivity of the salience network and DMN, specifically in its ventral nodes.

## 2. Anxiety Regulation and Its Neural Bases: The Cognitive View

In the previous paragraph, we explored some basic psychodynamic concepts on anxiety generation and its basic neural mechanisms. In this paragraph, we explore the dynamics behind anxiety regulation, dysregulation, and their neural bases. One powerful way to characterize psychological disorders is to understand how individuals regulate or fail to regulate anxiety and other affective reactions [2,3,4,21,22,23,24,25,26]. The concept of emotion regulation includes the neurocognitive process by which individuals manipulate the strength (intensity), onset, and manifestation of their emotions [27]. According to the cognitive model of emotion regulation [27], emotions are generated through the following steps: (i) people face a problem, or a negative or positive experience; (ii) they focus on a particular aspect of the experience; (iii) they shape that experience; (iv) an emotional response arises, which has an adaptive action tendency, a feeling, and physiological arousal; and (v) finally, individuals adjust the response. According to this model, dysregulation happens if the patient lacks or fails to adopt an appropriate regulatory strategy. Thus, a psychotherapist might point out more effective regulatory strategies for a patient in need. Following this principle, cognitive behavioral therapy techniques (CBTs) include intentional strategies that focus on cognition and attention domains for individuals who struggle with dysregulated emotions [28,29]. Evidence shows that in this way, individuals can acquire emotion-regulation skills [30].

In the last twenty years, the neural mechanisms underlying basic cognitive regulatory processes from a cognitive perspective have been studied [4,28,31,32]. Both the inferior parietal cortex (IPC) and the dorsolateral prefrontal cortex (dlPFC) play an important role in controlling attention and working memory [33]. The anterior cingulate cortex (ACC) is commonly linked to ongoing processes’ control and monitoring [34]. The ventrolateral prefrontal cortex (v1PFC) seems to be associated with inhibiting inappropriate responses [35] and selecting goal-driven ones [36,37]. The amygdala is a neural region that supports the processing of external and internal emotional stimuli [38,39] and negative stimuli [40].

Only recently, Grecucci and colleagues [41] have started studying more complex forms of regulation of socially cued emotions in interpersonal situations. The ultimatum and dictator games were used to elicit interpersonal emotions such as anger and moral disgust. The insula, a brain area responsible for aversive responses aroused by unfair behaviors in both tasks, showed decreased activation during emotion modulation [42,43,44].

Besides magnetic resonance imaging results, emotion-regulation-related processes are also reflected in changes in electrocortical potentials (event-related potentials). A recent experiment showed an increase in a negative fronto-central component named stimulus-preceding negativity (SPN), when individuals implemented a regulation strategy [45]. When participants applied the regulation strategy to emotional stimuli, a decrease in the late positive potential (LPP) elicited by emotional stimuli was repeatedly found [45,46,47]. Last but not least, the regulation strategy also affects brain oscillatory activity, with a modulation of theta frequencies [48], interpreted as a cortico-subcortical interaction between modulating and modulated brain regions, but also as a sign of increased relaxation after having regulated emotions.

The studies described so far investigated emotion regulation through the lens of cognitive models, focusing on the regulation of emotions themselves. According to this perspective, the preferable approach to deal with dysregulated emotional states consists of actively regulating them in the context of specific protocols [49] or trainings [50,51]. On the contrary, from a psychodynamic perspective, emotion dysregulation is strictly associated with anxiety (that can affect emotion by replacing it or covering it) or with secondary-defensive affects. This approach, that takes into consideration the role of anxiety in regulating emotions, is still little investigated and understood.

## 3. Problems with the Cognitive View of Anxiety Regulation

Based on appraisal theory [52,53], Gross’ [27] emotion regulation framework suggests that the appraisal of a stimulus generates an emotional response. Building on this, cognitive behavioral therapies focus their attention on cognitive factors to regulate emotions. According to this point of view, dysregulated emotions arise because of an inappropriate or ineffective use of regulatory attentive, cognitive, and behavioral strategies [54,55]. However, this is not consistent with neuroscientific findings (see [4,23,24,56] for a discussion). When emotional experience unfolds, neurobiological precedence over cognition is observed in terms of temporal brain-related dynamics. A stimulus triggers a subcortical response, milliseconds before cognitive elaboration occurs in the frontal and occipital cortical areas [57]. First, the amygdala is activated and it triggers the body’s emotional reactions, as we have seen in the previous section. Milliseconds later, the amygdala sends a message to the cortex. Then, cognition occurs (although several studies showed that it is not necessary for an emotional reaction to happen). Emotion processing occurs before cognitive processing in terms of anatomical circuitry. Indeed, connections are observed between emotional structures and perceptual systems, but not among cognitive structures and perceptual systems [56,58].

If cognition occurred first, we would expect cognitive strategies to be effective in top-down emotion regulation. On the contrary, research findings (but also clinical observation) indicate that cognitive strategies may fail to regulate emotions when emotional activation is high. For instance, research [59,60] shows that participants—if asked to choose between distraction or reappraisal by taking into account specific situations—tend to deploy distraction only for high-intensity stimuli, whereas they prefer to use reappraisal for low-intensity emotional stimuli. These results reveal the lessened efficacy of cognitive regulation strategies for more stressful events, the kind that patients struggle with in the real world [58]. Neurobiologically, overactivated emotion-related regions dampen the activation of the prefrontal cortex [61,62]. Thus, when emotion is excessively dysregulated, other strategies rather than cognitive ones must be used.

## 4. Toward a Psychodynamic Model of Anxiety Regulation: Concepts and Possible Neural Bases

Now, we focus on how experiential and psychodynamic therapies [63,64,65,66] and affective neuroscientific discoveries [56,67,68] can offer a different perspective on emotion regulation and dysregulation. This alternative view was named “experiential dynamic model of emotion regulation” (EDER) [2,4,23].

According to this perspective, emotions are evolutionary products generated to motivate actions that can change while the situations unfold [1,2,11,12,63,65,66,69,70,71,72,73,74,75,76,77]. Emotions get dysregulated because they become associated with conditioned anxiety or because they are paired with secondary emotions caused by defenses (e.g., defensive affects) [66]. When the therapist encounters excessive anxiety, anxiety needs to be regulated for the underlying emotions to emerge. In contrast, when defensive affects cover underlying emotions, the therapist needs to block the defense to deactivate defensive affects, so emotions can emerge. In this paper, we focused our attention on the role of anxiety in emotion dysregulation.

The experiential dynamic emotion regulation model (EDER) [2,23] claims that emotional stimuli trigger responses present at birth [76] with innate adaptive action dispositions [72] and facial expressions [71]. These occur before cognition (neuroanatomical and temporal primacy) [12,76]. Neuroscientific findings [56,76,78] suggest that subcortical brain regions implicitly and automatically generate emotions with specific features (proportionate intensity and duration to the event) to make sense of what is going on (see Section 1). Specific biological mechanisms automatically regulate emotions [58].

As a result, emotion regulation does not necessarily require intentional control [2,23]. Emotions are turned into healthy acts [79,80] and—the moment the adaptive behavior has accomplished its evolutionary function—they return to the base point [66]. Indeed, emotions are not dysregulated by nature [2]. Unregulated anxiety plus emotion results in dysregulation. Following anxiety regulation, emotions can emerge and be processed.

The therapist pursuing the experiential dynamic model handles emotional dysregulation by regulating anxiety. After that, the therapist encourages the patient to fully experience the emotion without increased anxiety or defenses [63,65,66,70,74,81,82]. By experiencing their emotions, individuals can focus on the associated bodily sensations to promote emotional self-awareness, considered by some authors as a more valuable approach than cognitive techniques [83,84,85].

From a neural point of view, there are a few studies trying to understand emotion regulation from a more dynamic experiential point of view rather than the standard cognitive view. An example is the study of neural substrate of acceptance-based regulation—defined as a welcoming non-judgmental attitude toward emotions and thoughts (in fact, the contrary of cognitive control) [86,87,88,89]. Another example is the study of the neural correlates of implicit forms of emotion regulation, such as spontaneous emotional avoidance [90,91]. In all these studies, brain areas involved in cognitive control have been not detected, whereas modulation of semantic and default system structure seems to constitute a more specific neural substrate of experiential emotion regulation [92,93].

Since different brain areas are involved in cognitive vs experiential dynamic regulation, Grecucci and collaborators [4], hypothesized that they rely on two different neurobiological processes. *Memory extinction* may be the core biological mechanism behind cognitive regulation and cognitive therapies. Memory extinction (momentary suppression of anxiety-eliciting memories), may be mediated by top-down control mechanisms. Indeed, experimental results suggest that cognitive strategies regulate subcortical areas responsible for emotion activation (amygdala, insula, etc.) via top-down modulation of the prefrontal cortex. Consistent with this view, studies of neurobiological changes due to a cognitive therapy have shown increased activation in the frontal cortical regions, such as the prefrontal cortex and anterior cingulate cortex, and decreased activity in subcortical regions (see [94,95] for anxiety disorders, but also [96] for depression, and [97] for a more general review). Dynamic and experiential approaches [2], are not based on strategies supposed to top-down regulate subcortical areas. Instead, they promote the re-experiencing of warded-off emotions (sometimes called “corrective emotional experience”). This may facilitate another neurobiological process known as *memory reconsolidation*, that holds the power to erase the original maladaptive emotional learning (see [4] for details). Neurobiological studies on acceptance (a strategy included in experiential dynamic therapies) confirm the lack of prefrontal top-down control areas during this type of regulation. Instead, they show modulation at a subcortical level (bottom-up process).

## 5. Principles of Experiential Dynamic Techniques for Regulating Anxiety

Dynamic experiential treatments [21,65,66,81,82,98,99,100] differentiate primary emotions from dysregulated affect states (DAS). Primary emotions result from subcortical neuroperception of the surrounding environment—the experience of stimuli in the real world [12,56,65,66,76,77,78,81,82,101]. Instead, DAS result from emotions associated with excessive conditioned anxiety [65,66,81,82].

These considerations have important consequences for psychotherapy practice. First, to analyze the patient’s relationships that trigger his/her problems, therapists should differentiate primary emotions from anxiety, and then regulate anxiety, not the emotions themselves.

DAS resulting from emotions paired with unregulated anxiety require anxiety regulation. Emotion regulation techniques used by experiential dynamic therapists reduce patients’ anxiety until they are able to experience their feelings without excessive anxiety [65,66,102]. To avoid future relapses—which may constitute a risk in cognitive therapies (see [103,104])—experiential dynamic therapists take an extra step. They help patients to bear higher levels of the feeling without anxiety and defenses [65,66,81,82]. Then, higher levels of feelings will no longer trigger the defenses that cause patients to present problems and symptoms.

Thus, the therapist explores the emotion that the patient feels toward a specific person. Then, as the feeling rises, the therapist assesses anxiety and defenses constantly. If a DAS occurs, the therapist either regulates anxiety or deactivates a defense. Once the DAS disappears, the therapist can safely explore feelings again. In this process, the patient can eventually feel his previously avoided feelings and channel them into an effective action [79,80,105]. Then, his/her feelings no longer trigger the anxiety and defenses that previously caused his/her DAS.

Defenses are unconscious forms of emotion “dissociation” that prevent forbidden feelings from entering awareness and causing anxiety [106,107]. Some defenses keep feelings out of awareness, thus reducing anxiety. For example, when using a defense mechanism such as intellectualization, patients will be aware of their thoughts, but not their feelings. Thus, their anxiety will decrease. If the therapist blocks the defense and invites the underlying feelings, patients’ anxiety will increase. Now, the therapist and patient can face the avoided feelings that triggered anxiety in the first place. Here, anxiety functions as a “signal” that avoided feelings are rising [106,108]. Thus, signal anxiety indicates when the patient is getting closer to previously avoided material. By helping the patient to see and let go of his defenses and regulate anxiety, the resulting dysregulation disappears. Then, the therapist helps the patient to experience the feelings underneath the anxiety. Once patients have this level of affect tolerance, they can channel their feelings into adaptive actions. Then, feelings can once again achieve their original evolutionary purpose: adaptation.

In sum, although not systematically, in the present paper, we reviewed some aspects related to emotion regulation and dysregulation, and presented two main models: the cognitive model [27] and the experiential dynamic model [2,3,4,23]. We focused on the experiential dynamic model that is derived from affective neuroscience findings and from psychodynamic and experiential psychotherapy approaches that focus on emotion regulation. This model claims that non-conscious physiological mechanisms activate emotions without conscious awareness. In response to external and internal stimuli, the brain generates non-conscious affects [12]. Emotions have physical properties, such as duration and strength, which correlate with the intensity of the stimulus. Emotions are activated so that they can be converted into an adaptive action. They are not inherently dysregulated. Emotional dysregulation results from dysregulating anxiety and defenses. It does not result from the lack or the weakness of regulatory objectives, as held by the CBT perspective. According to this approach, dysregulation arises from emotions associated with discharged excessive conditioned anxiety. Frederickson et al. [2] suggested a distinction between DAS that are feelings paired with excessive anxiety, and DAS that are defensive affects resulting from defenses. These emotional states must be regulated [2,3,23]. At excessive levels, anxiety shuts down the prefrontal cortex, damaging reality testing and negatively affecting cognition. When anxiety is regulated, reality testing (and PFC) is restored, and cognitive work (in CBT or psychodynamic settings) is possible. In sum, the therapist helps the patient to experience feelings triggered by real stimuli and to regulate anxiety when DAS result from the pairing of an emotion and excessive anxiety (see [66] for further detailed information). Through these strategies, the patient learns to bear feelings without the anxiety and defenses that create DAS. While mitigating DAS, the therapist upregulates emotions until the patient completely faces the hitherto avoided affects without DAS. The patient will feel relief and learn to channel emotions into effective and adaptive actions.
